# Membranous Stomatitis Complicating Pneumonia—Eight Cases Reported*Read before the Texas State Medical Association, May 7, 1902.

**Published:** 1902-08

**Authors:** J. M. Frazier

**Affiliations:** Belton, Texas


For Texas Medical Journal.

### Membranous Stomatitis Complicating Pneumonia—Eight Cases Reported.*

BY J. M. FRAZIER, M. D., BELTON, TEXAS.

I desire to submit for the purpose of record in the Transactions of this Association eight cases of membranous stomatitis, occurring in my own practice, coincident with and seriously complicating lobar or croupous pneumonia.

Two of these cases were seen in 1900, two in 1901, and four in February and March of this year. All the patients were adult young women. All were residents of a female boarding school in Belton. The pneumonia was of the croupous or lobar form. In two cases both lungs were involved. They were all typical cases of pneumonia, ushered in with chill with physical signs so marked that an early diagnosis was easy. In every case anorexia, nausea and vomiting were prominent symptoms from the first—and persisting in most of the cases to the crisis.

The temperature in all the cases was high, ranging from 103° to 106°. The pulse rate varied in different cases, in the graver cases being excessively rapid, 130 to 140. The crisis of the pneumonia was marked and uniformly came on the seventh or eighth day.

The membrane began to develop on the third day of the pneumonia. This I noted particularly in the last three cases. Previous to this I had not noticed its appearance, until my attention was called to it at about the time of the crisis. I am now satisfied if I had examined the mouth I would have found it beginning to appear in all the cases earlier. The membrane, when observed early, was first seen on the arch of the palate, on one or both sides

*Read before the Texas State Medical Association, May 7, 1902.

of the uvula. Extending by patches and not contiguously to the uvula, cheeks, tongue, roof of the mouth and under the tongue. At it resembled a thin film of spider web lace, or like the lichen growing on a damp brick wall, of a grayish white color, gradually thickening from day to day and requiring uniformly from five to 8 days to mature and exfoliate.

It was firmly attached, and could with difficulty be removed, and when forcibly detached with forceps left a red, raw and bleeding surface. When matured it was a thick, tough leathery grayish white membrane, loosening at the margin and then easily removed. There was in each case considerable soreness complained of, but not excessive. There was in each case marked pharyngeal catarrh, but no membrane noticed on the back wall of the pharynx. The tongue was swollen. There was but little apparent involvement of the glands. There was difficulty of expectorating and secretions were gotten rid of by gagging and vomiting, or by mechanically wiping them out. There was a distinctly bad odor to the breath in all cases. In two cases this was almost gangrenous. These two cases had decided septic symptoms, persisting a week after the typical pneumonia crisis. Much of the membrane was swallowed and appeared in the discharges from the bowels unchanged.

In four of the cases the urine was examined and found to contain albumen, alkaline in reaction, and specific gravity varying from 1005 to 1015.

The pneumonia in all the cases had the usual symptomatic and supportive treatment, milk forming the staple of food.

The last four cases, occurring this year, had ten to fifteen drop doses of carbonate of creosote every four hours.

Delirium was present in two cases, both cases of double pneumonia with septic symptoms. The mouth was cleansed with cotton swabs, sprays and washes freely and frequently, all sorts of germicidal and antiseptic agents being used. There was never any evidence of contagiousness from patient to nurse or other patient. I regret that I did not attempt to inoculate some of the lower animals with the membrane to determine more positively this point. Three of the cases had, during the second week of their illness, suppuration of one or both ears, preceded by pain and perforation of the drum membrane.

All recovered, the two worst cases requiring a month for convalescence, and both complained of difficulty of co-ordinating muscular movements. One had difficulty of swallowing and a tendency to regurgitate liquids into the nose. No other evidence of even partial paralysis was present.

As to the etiology of these cases, I am unable at present to satisfactorily explain them. I had many other cases of pneumonia at the same time, and several in the same buildings, without the membrane.

The milk, food and water supply was the same, and careful inspection failed to develop any local cause to account for it.

I had Drs. Hudson, Ghent and Embree, of Belton, and Woodson and McCelvy, of Temple, and other physicians see the cases at various times. None of them had previously observed a similar condition or complication. I have been unable to find any published reports of such cases. I believe it is a true parasitic membrane. I have submitted specimens to Dr. Allen J. Smith, pathologist of the Medical Department of the State University. He has taken a kindly interest in the matter, and is making cultures, and I hope will be able to identify the etiological organism.

Believing the cases to be unique and worthy of record, I submit this crude report.

---

GALVESTON, TEXAS, May 7, 1902.

*Dear Dr. Frazier.*

I am unable to bring you my report of your bacteria, and at this late hour can only condense the report.

Two germs were recovered from the false membrane submitted to me. One was a bacillus, sometimes occurring in chains, usually isolated. It measures 2½-4 m. in length by a little less than 1 m. in thickness. It is motionless; grows well at body temperature, less well at temperature of the room. Grows well in agar and on blood serum, producing a white, rather dry surface growth. flat, in rounded colonies with slightly fringed edges. No liquefaction of the serum. Does not liquefy gelatine. Stains with the ordinary laboratory aniline; stains not well with Gram's, fairly with Loeffler's blue. Produces a slight turbidity in bouillon, diffuse, without production of gas. Is aerobic. I regard it as a pseudo-diphtheritic bacillus of a somewhat atypical variety. No inoculation in animals done.

The other germ is a diplococcus, not the pneumococcus, but I am unable for lack of time at present to carry it out to determination.

I have been so much occupied with my class and other duties, and working in the other laboratories than the bacteriological during this latter part of the term (and hence not able to keep track easily of the bacteriological cultures), that I have failed to complete this work as I wished. I will as soon as I am free, however, complete the work to your and my satisfaction.

Very sincerely,

ALLEN J. SMITH.

